# Research on dynamic response characteristics of normal fault footwall working face and rock burst prevention technology under the influence of the gob area

**DOI:** 10.1038/s41598-023-45904-8

**Published:** 2023-10-31

**Authors:** Lianhai Tai, Chong Li, Shitan Gu, Xiaoxiao Yu, Zhijun Xu, Lei Sun

**Affiliations:** 1 School of Mines, China University of Mining and Technology, Xuzhou, 221116 China; 2 MOE Key Laboratory of Deep Coal Resource Mining, China University of Mining and Technology, Xuzhou, 221116 China; 3 School of Energy and Mining Engineering, Shandong University of Science and Technology, Qingdao, 266590 China

**Keywords:** Petrology, Structural geology

## Abstract

To study the effect of mining dynamic response characteristics on the footwall working face of the normal fault under the influence of the gob area, theoretical research, indoor experiment, and numerical simulation are adopted to analyze the stress manifestation characteristics, overburden movement, and energy evolution characteristics during the process of mining. The results show that: (1) In the process of mining toward the fault, the working face shows the change characteristics of “stable-activation mutation-final stability”. At 20 m from the fault, the arch structure of the working face was damaged, fissures appeared near the high fault fracture zone, and the displacement of the overburden rock increased significantly; (2) the maximum value was reached at 4–8 m from the coal wall, and the superposition of tectonic stress and mining stress led to the concentration of the stress and energy accumulating on the top plate near the fault, and the data close to the gob area were even larger; (3) If the plastic damage zone of the high-level rock layer on the hanging wall and footwall of the fault appears to have a wide range of penetration, and the area formed between the shear displacement curve of the fault plane and the X-axis appears to have a significant enhancement, it is considered that the fault has been activated; (4) The size of the coal pillar of the fault is determined to be 40 m, and combined with the pressure unloading technique of the variable-diameter drilling hole, the validation is carried out through the micro-vibration monitoring, and the results of which can be used as a reference for the safety of the working face under similar conditions.

## Introduction

With the high-intensity mining of coal in recent years, the rock burst has also been widely emphasized due to its characteristics such as suddenness and disastrousness^1^. Numerous studies have shown that geological structures are often accompanied by stress concentration^2,3^, which is easier to induce rock burst accidents^4,5^. The fault is a common geological structure, because of the characteristics of severe fragmentation and complex lithology, during the mining, mining stress, and tectonic stress superposition. Causing the hanging wall and footwall to slip, the energy is suddenly released, forming rock burst^6–10^.

Domestic and foreign scholars' research methods on fault structure mainly include theoretical analysis, microseismic monitoring, and numerical simulation^11–13^. Cai^14^ proposed a conceptual model of mining arrangement and fault activation and confirmed the mechanism that the superposition of dynamic and static load stresses leads to the activation of faults and induces the rock burst. Sainoki^15,16^ used numerical simulations to analyse the characteristics of fault stiffness, friction angle, and the gob area with the shear displacement of the fault. Kong^17^ analyzed the change of positive and shear stresses in the fault zone and classified the fault sliding mechanism into three categories. Li^18^ analyzed the activation of the fault by shifting the peak of the abutment pressure to the fault when the working face mined to the fault and the abutment pressure continues to decrease after crossing the fault, and eventually stabilizes. Lu^19^ used FLAC^3D^ to numerically simulate the fault sliding under the influence of the mining action of the adjacent working face and proposed a fault-sliding mechanism induced by the mining action. Jiang^20^ analyzed the seismic fault-slip behavior of a long wall working face passing through the main fault and evaluated the probability of fault-slip impacts using the peak support stress and peak particle velocity (PPV) obtained from the model results. Xing^21^ simulated using the UDEC software and analyzed the fault-affected roof of the mining roadway stability.

In addition, many scholars have also carried out extensive research on fault activation using physical similarity simulation^22–25^. Wang^26^ used indoor experimental studies to confirm that a sudden change in strain is a precursor of fault sliding and proposed that during the mining. It is also proposed that the influence of the overburden rock movement in the gob area between the fault zones should be paying attention to during the mining. Dou^27^ compared and analyzed the influence of the presence or absence of tectonics on the deformation of the roadway, and concluded that the tectonic stress field promotes the deformation of the roadway, which guides the optimization of the roadway layout. Bornyakov^28^ analysed the characteristics of stress change on the fault plane during fault slip and confirmed that the possibility of fault activation is significantly increased when the working face is mined to the fault. Zhang^29^ creatively proposed a new type of solid-like fluid and carried out a feasibility simulation analysis to study the behavior of fault activation. Wang^30^ analyzed the characteristics of overburden movement with the help of a physical similarity simulation test and determined the intrinsic connection between fault activation and rock burst.

Previous studies have mainly focused on the mineral pressure law and the characteristics of overburden rock movement when fault activation occurs. There are few reports on the intrinsic connection between mining and the stress change and energy distribution of the fault plane under the influence of the gob area. Therefore, in this paper, the response characteristics of the fault during the mining of the working face on the footwall of the normal fault under the influence of the gob area are investigated, and the physical similarity simulation and numerical simulation complement each other and put forward the measures to prevent the rock burst of the fault area. The results of the study can provide a reference for the prediction of rock bursts and the classification of hazardous areas under similar conditions.

## Engineering background

**Figure 1:**
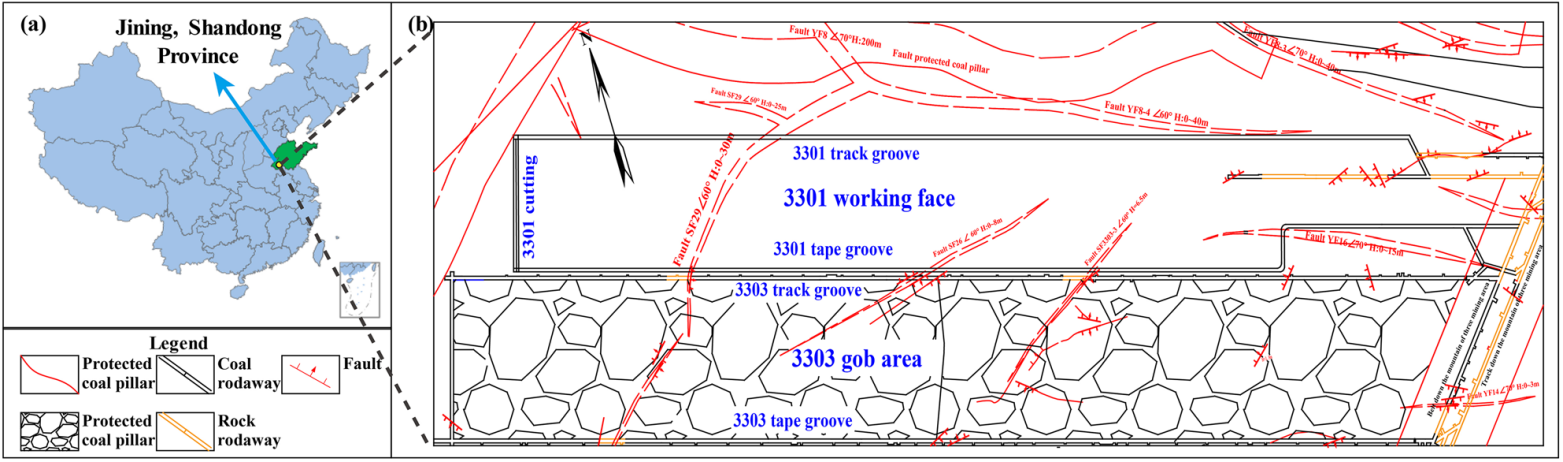
Location and faults distribution of study area.

The 3301 working face of Yiqiao coal mine is fully mechanized caving coal face, the − 335 mining level, the depth of the 3301 working face is 537.3–583.5 m, with an average of 560.4 m, and the layout of the working face is as shown in Fig. 1. The south side of 3301 working face is 3303 gob area, which was mined in 2017, and the width of coal pillar between two working faces is about 5 m. The coal seam’s average angle of 13°. The width of the working face is about 100 m, the thickness of the coal seam is 4.0 m, the height of the coal cutter is 2.4 m, and the height of the coal release is 1.6 m. According to the exploration and 3D seismic geological data, the working face is cut by fault SF29 (0–20 m), and the dip angle of the fault is 60°.

## Materials and method

### Laboratory experiment

#### The experimental program

The study was carried out using a two-dimensional similarity simulation test bed, with frame specification parameters of 1.90 m × 0.22 m × 1.20 m. According to the dimensions of the similarity simulation test frame and the mining range of the coal seam, the uniaxial compression test of several groups of rock specimens was utilized, and after repeated tests and comparisons, it was determined that the model geometrical similarity ratio was 1: 200, the bulk density similarity ratio was 1: 1.76, and the strength similarity ratio was 1: 300. The main materials are filler as well as binder, the former is usually selected from river sand, talc, etc., while the binder mainly uses gypsum, calcium carbonate, and other materials. This experiment aims to better study the movement characteristics of the overburdened rocks, and gypsum is used as the binder, an appropriate amount of calcium carbonate is added, river sand is used as the filler, and the appropriate amount of mica powder is laid on the contact surface of each rock layer^24,31,32^. The variability of simulated objects and proportions can be expressed with the different ratios of materials, and the material depletion coefficient is taken to be 1.1 due to the possible loss of materials during the test. The specific parameters of each rock layer are listed in Table 1.

**Table 1:** Model material ratio, materials, and laying level.

Lithology	Density		Compression strength		Elastic modulus		Material ratio
	Prototype (kg·m^−3^)	Model (kg·m^−3^)	Prototype (MPa)	Model (KPa)	Prototype (GPa)	Model (MPa)	
Fine sandstone	2550	1448	78.63	26.21	6.49	2.16	764
Siltstone	2450	1392	56.20	18.73	5.73	1.91	755
Mudstone	2150	1221	47.34	15.78	5.02	1.67	864
Medium sandstone	2650	1505	66.42	22.14	6.18	2.06	773
Coal 3#	1440	818	15.86	5.29	2.35	0.78	864
Sandy mudstone	2150	1221	32.42	10.81	3.06	1.02	873

The laying of the model can be divided into five steps: weighing the material, adding water and mixing it well, loading the model, laying the mica powder, and removing the excess material. The total height of the test bed is 1.1 m, and the thickness of the roof about the test simulation is 0.8 m, corresponding to the actual simulation height is 160 m. However, the physical model cannot fully simulate all strata of the prototype (mining depth of 560 m). According to the buried depth of 3301 working face and the ground stress lateral pressure coefficient of 1.12 measured on site, the similar model was loaded vertically (800 kg weights were added at the top) and horizontally (both sides of the model were loaded uniformly by using oil cylinders.

**Figure 2:**
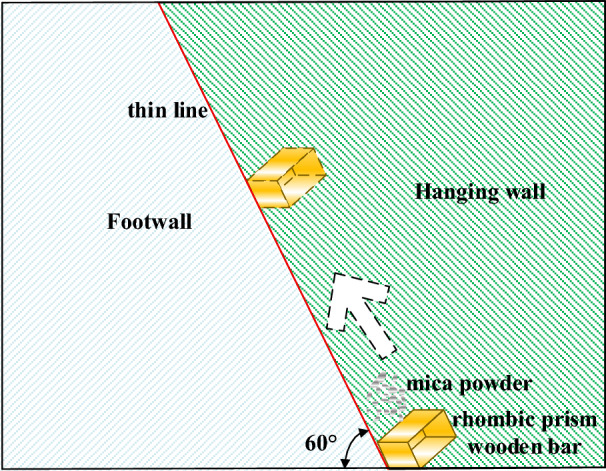
Schematic diagram of fault zone formation process.

Before the model is laid, a preliminary design of the fault pattern is carried out, and a clamp is fixed on the upper part of the rack and the lower part of the rack respectively, a thin line is clamped and tightened, and the angle between the thin line and the horizontal plane is strictly controlled to be 60°, to facilitate the subsequent determination of the basic direction of the fault zone. Design a rhombic prism wooden bar, the size of the wooden bar is 22 cm × 2 cm × 2 cm, and the wooden bar and the horizontal plane of the test bench form a 60°. In the process of laying the model, the wooden bar is pressed against the bottom of the test bench, so that one side of it is close to the thin line, and manually clamped and fixed to ensure that the plane and the thin line are in close contact with each other, without changing the shape of the thin line. After laying the rock layer on the footwall of the fault, then lay the rock layer on the hanging wall of the fault, and then take out the wooden bar and fill the mica powder in it, which is used to simulate the fault zone, and the thickness of the laying is 20 mm^30^. With the laying of the rock layer, the wooden bar was cycled upwards until the modeling was completed, details as shown in Fig. 2.

The model was laid for 15 days before mining, the daily footage of excavation at the project site was 2 m, and the model was excavated in two days as a unit; to ensure that the overlying rock strata collapsed sufficiently after excavation, the excavation was carried out by 2 cm. To reduce the influence of boundary effects, a 15 cm coal pillar was reserved at each end of the model. The movement of the overburden was recorded with a high-definition digital camera. The strike length of the working face in the model is 380 m, and the final mining length is 320 m.

#### Monitoring program

**Figure 3:**
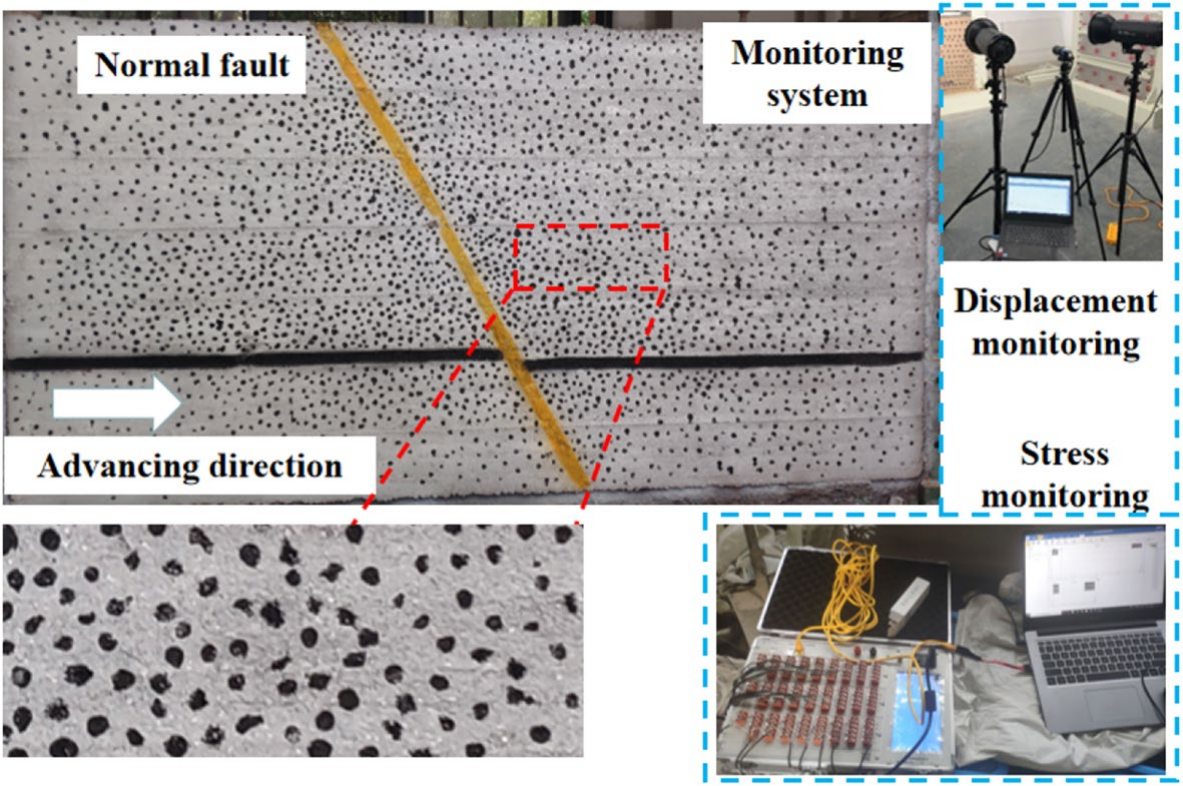
Simulation model of similar materials.

**Figure 4:**
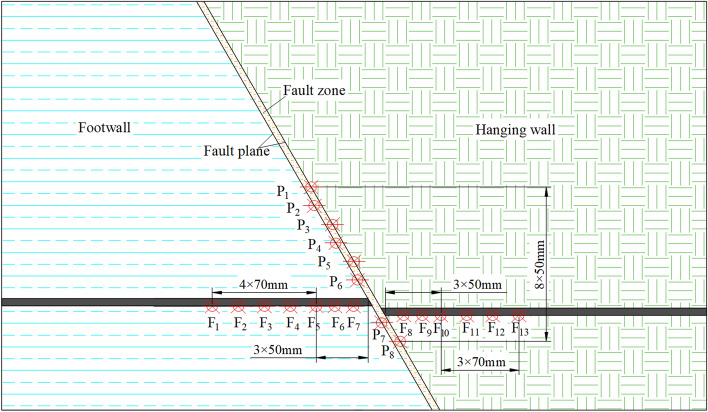
Model stress sensor layout.

1. To monitor the deformation of the overburdened rock during the excavation of the model, irregular scattering spots were added on its surface and encrypted in the roof and fault zones. The deformation of the model was monitored by the digital scattering system, and a photograph of the scattering measurement point of the specimen was collected every 10 min. The measurement point arrangement and equipment are shown in Fig. 3.
2. To monitor the rock stress during model excavation, the sensors were buried inside the model during model laying, and the stress changes at the measurement points were measured by the DH3815 static strain gauge with a sampling frequency of 12 Hz. Thirteen sensors are arranged in the bottom plate to monitor the change of coal body abutment pressure, and eight sensors are arranged in the fault plane to monitor the change of stress in the fault plane, as shown in Fig. 4. Before excavating the coal seam, the sensors are “zeroed”, and the data recording interval is 10 s.

### Numerical simulation

#### Parameters of 3301 working face model

**Figure 5:**
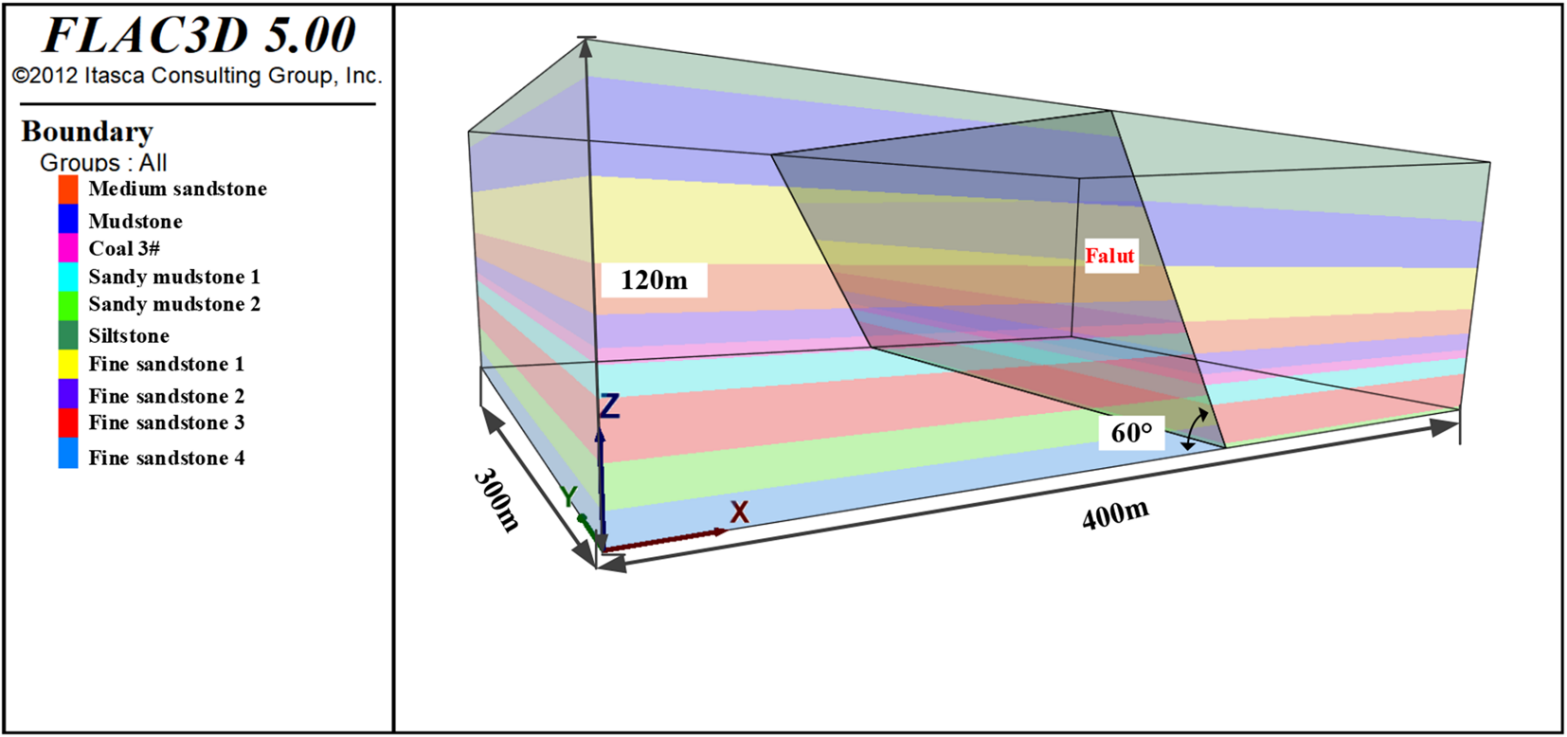
Schematic diagram of the numerical model of 3301 working face.

The model size X × Y × Z = 400 m × 300 m × 120 m, all rock layers were divided into 3 × 4 × 2 m^3^ units, and the working face was mined along the X-axis direction. The overlying rock layer’s volumetric weight is 25 kN/m^3^. Displacement constraints are imposed on the lateral and lower surfaces of the model. A uniform load of 14 MPa was applied to the top of the model, and the stresses in the X and Y directions were 12.6 and 11.8 MPa, respectively, in combination with the ground stress test data. 50 m boundary coal pillars were left to eliminate the influence of the model boundaries on the simulation results, and the numerical modeling schematic diagram is shown in Fig. 5.

The values of physical and mechanical parameters of coal and rock seams are taken concerning the actual geological data of 3301 working face and combined with other similar geological conditions, and the detailed rock mechanical parameters are shown in Table 2.

**Table 2:** Table of rock mechanical parameters.

Lithology	Thicknesses (m)	Densities (kg*m^-3^)	Bulk modulus (GPa)	Shear modulus (GPa)	Cohesive (MPa)	Internal friction angle (°)
Siltstone	8	2450	2.3	1.8	5.6	40
Shale	22	2150	4.1	3.9	3.2	38
Fine Sandstone 1	20	2550	12.3	10.8	6.6	42
Medium Sandstone	12	2650	13.6	12.1	6.6	40
Fine Sandstone 2	8	2550	14.5	13.7	7.3	42
Coal 3	4	1440	1.83	1.53	2.7	38
Sandy Mudstone 1	8	2150	4.2	3.8	3.2	38
Fine Sandstone 3	16	2550	12.3	10.8	6.6	42
Sandy Mudstone 2	12	2350	4.2	3.8	3.5	38
Fine Sandstone 4	10	2550	14.5	13.7	7.3	42

Regarding the fault plane parameters, normal stiffness: k_n_ = 2 × 10^9^ N/m; tangential stiffness: k_s_ = 2 × 10^9^ N/m; internal friction angle is set to 21°; cohesion is set to 0.5 MPa.

In the excavation process of a deep-buried coal and rock mass, the tiny texture that already exists inside the coal and rock mass will continue to expand, and its strength will continue to decrease. Combined with the post-peak morphology of the rock stress–strain curve during the loading process, the cohesion and internal friction angle of the coal seam, roof, and floor rock layers^33–35^ were re-assigned to realize the adjustments to the modulus of elasticity and Poisson's ratio, and the specific strain-softening parameters are shown in Table 3.

**Table 3:** Strain softening model parameter settings.

Rock layer	Lithology	The cumulative plastic shear strain value	Internal friction angle (°)	Cohesion (MPa)
Floor	Sandy Mudstone 1	0	38	3.2
		0.05	36	2.7
		0.1	34	2.2
Coal	Coal 3	0	38	2.7
		0.05	36	2.3
		0.1	34	1.9
Roof	Fine Sandstone 2	0	40	5.6
		0.05	37	5.0
		0.1	34	4.4
	Medium Sandstone	0	42	7.3
		0.05	40	6.5
		0.1	38	5.7

#### Simulation and monitoring program design

**Figure 6:**
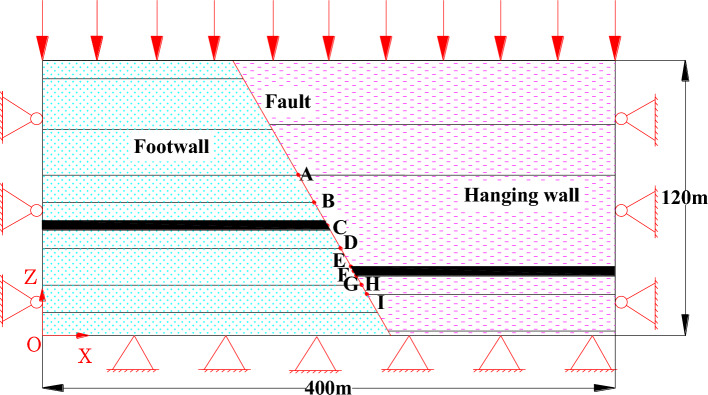
The SF29 fault plane measurement point location map.

The width of the 3301 working face and 3303 working face is set to 100 m, using the method of segmental excavation solution, 3303 working face is excavated once and mined to the fault SF29. The 3301 working face is divided into 9 times of mining to the fault, and the final mining to the fault 150 m. A total of 9 groups of monitoring points A-I were set up to monitor the changes in normal stress, shear stress, normal displacement, and shear displacement during the process of mining to the fault plane, and the arrangement of the monitoring points is shown in Fig. 6.

To clarify the stress and energy evolution law in the process of mining towards fault SF29 in 3301 working face under the influence of the adjacent gob area, the measurement lines were arranged in the roof near and in the middle as well as far away from the 3303 gob area, which was recorded as measurement line L_C_, L_M_ and L_F_, and measurement points were set up at intervals of 2 m in each line, and finally, each measurement line had 50 measurement points. The three lines always keep monitoring the data of the direct roof within 100 m in front of the coal wall.

#### Methods for calculating the elastic strain energy density of rock masses

The strain energy density (expressed in terms of stress, strain) of an elastomer in a three-dimensional stress of stress is derived in elastic mechanics as:

1 $$ U = \frac{1}{2}\left( {\sigma_{x} \varepsilon_{x} + \sigma_{y} \varepsilon_{y} + \sigma_{z} \varepsilon_{z} + \tau_{yz} \gamma_{yz} + \tau_{xz} \gamma_{xz} + \tau_{xy} \gamma_{xy} } \right) $$

where U is the strain energy density of the elastomer, KJ/m^3^; $\sigma_{x} ,\sigma_{{\text{y}}} ,\sigma_{z} ,\tau_{yz} ,\tau_{zx} ,\tau_{{x{\text{y}}}}$ are the 6 stress components at a point within the elastomer; $\varepsilon_{x} ,\varepsilon_{{\text{y}}} ,\varepsilon_{z} ,\gamma_{yz} ,\gamma_{zx} ,\gamma_{{x{\text{y}}}}$ are the 6 strain components at a point inside the elastomer.

Considering the rock mass as isotropic, the relationship between stress and strain at a point within the elastomer (the physical equation) is given by the generalized Hooke's theorem:

2 $$ \left\{ {\begin{array}{*{20}c} {\varepsilon_{x} = \frac{1}{E}\left[ {\sigma_{x} - \mu \left( {\sigma_{y} + \sigma_{z} } \right)} \right],\gamma_{yz} + \frac{1}{G}\tau_{yz} } \\ {\varepsilon_{y} = \frac{1}{E}\left[ {\sigma_{y} - \mu \left( {\sigma_{z} + \sigma_{x} } \right)} \right],\gamma_{zx} + \frac{1}{G}\tau_{zx} } \\ {\varepsilon_{z} = \frac{1}{E}\left[ {\sigma_{z} - \mu \left( {\sigma_{x} + \sigma_{y} } \right)} \right],\gamma_{xy} + \frac{1}{G}\tau_{xy} } \\ \end{array} } \right. $$

where E is the modulus of elasticity; μ is the Poisson’s ratio; and G is the shear modulus of elasticity.

The relationship between E, µ, and G is:

3 $$ G = \frac{E}{{2\left( {1 + \mu } \right)}} $$

Substituting Eqs. (3) and (4) into Eq. (2) yields the elastic strain energy density expressed by the stress component as:

4 $$ U{ = }\frac{{\left( {\sigma_{x} + \sigma_{y} + \sigma_{z} } \right)^{2} - {2}\left( {{1} + \mu } \right)\left( {\sigma_{y} \sigma_{x} + \sigma_{z} \sigma_{x} + \sigma_{x} \sigma_{y} - \tau_{yz}^{2} - \tau_{zx}^{2} - \tau_{xy}^{2} } \right)}}{2E} $$

From the above equation, it can be seen that for the triaxial force roof, there is a positive correlation between the elastic strain energy density and the stress when the shear elastic modulus and Poisson's ratio are kept constant. Combined with the numerical simulation software, the fish language is written to reasonably investigate the energy distribution of the direct and basic roof during the cooperative deformation of the support system and the surrounding rock.

## Results

### Experimental results

#### Characteristics of overlying rock movement

**Figure 7:**
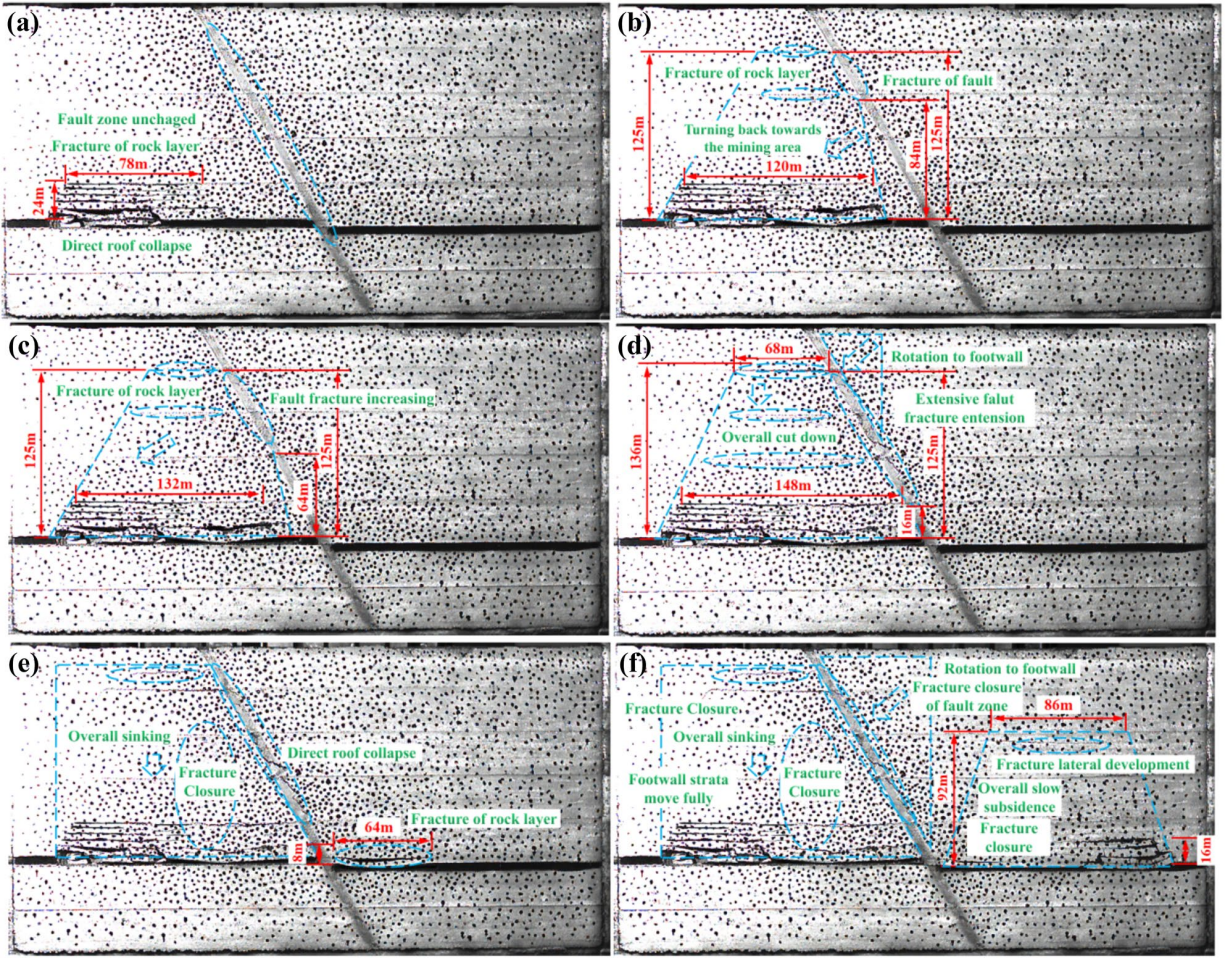
Overlying strata structure diagram at different distances between working face and fault: (**a**) L = 50 m; (**b**) L = 20 m; (**c**) L = 10 m; (**d**) L = 0 m; (**e**) L = − 100 m; (**f**) L = − 150 m.

For the working face arranged in the footwall of the normal fault, under the influence of fault cutting, the overburden structure has singularity characteristics, and the fault coal pillar size retention ultimately affects the structural evolution characteristics, and details are shown in Fig. 7. The distance between the working face and the fault (coal pillar size) is defined as L. When L = 50 m, Fig. 7a, the influence of mining on the fault is relatively weak, and the symmetrical arch structure is formed after the overburdened rock is broken. When L = 20 m, as in Fig. 7b, the arch structure is damaged to a certain extent, and symmetry cannot be realized. The fault fissure gradually realizes the closure, the relative slip of the hanging wall and footwall continues to decrease, and the overburden structure changes to the gob area. The low-level fault does not show obvious activation and faulted coal pillar lateral pressure arches cannot achieve continuous, the arch foot still has the corresponding bearing capacity, the main roof shows sufficient stability, and the fault coal pillar has a certain bearing capacity in this state, which plays a supportive role for the roof subsidence. In addition, at this time, the fault-blocking effect is increasing, high-stress zones appear in the coal pillar and near the fault, and the probability of occurrence of static-loaded rock burst is significantly increased. In the process of the working face continuously approaching the fault, the arch structure gradually disappears, and when L = 10 m, as shown in Fig. 7c, the fault plane continues to open, however, the fault plane at the main roof is still in a stable, which is known as the aggravation of fault local activation. It can be seen when the fault coal pillar is less than 10 m, the fault coal pillar has more serious plastic breakage, losing the bearing capacity, the fault plane is completely open, the low-level of fault shows the whole cut-off, and enters into the decompression zone, the pressure arch completely disappears. L = 0 m, as shown in Fig. 7d, the stress of the fault coal pillar is released, the stability continues to be reduced, rotational subsidence of the main roof, and the upper disk of the fault as a whole cut-off along the fault plane, with a loud sound. Indicating that the state of the mine pressure is particularly intense, prone to dynamic load-type rock burst, the state is known as the fault all activation.

When L = − 70 m, as shown in Fig. 7e, both the hanging wall and footwalls can be maintained in a relatively stable state, the mine pressure is weak, and the low fault plane is still open. As the mining, the area of direct roof overhang continues to become larger, and the phenomenon of leaving the layer gradually occurs. When the face was finally excavated for 320 m (L = − 150 m), as shown in Fig. 7f, the transverse fissures in the overburden of the hanging wall further developed, and the whole slowly settled, resulting in the gradual closure of the fissures of the direct roof and the main roof rock layer, the main roof broke and continued to develop, the fissures finally appeared in the rock layer of 92 m above the working face, and the horizontal span could be maintained at about 86 m. The high-level rock layers on the upper plate of the fault turned back to the gob area, the fault facture continued to close, which had an important influence on the footwall of the fault, and the rock layers were re-compacted and articulated again to form a new whole^36^.

In summary, at the early stage of mining, due to the relatively far distance between the working face and the fault, the collapse of the overburdened rock is similar to the performance when there is no fault structure, and the broken rock can still be extruded and formed to obtain the structure of masonry beams and rock beams. The broken overlying rock presents an arch structure and can realize the stress balance and the structure will be the front of the working face in the solid coal and the collapsed rock layer of the gob area as the front and back arches feet, and finally achieve stability. Since the main roof is composed of sandstone and medium sandstone, the strength of the rock layer is relatively high, which makes it impossible for the arch structure to realize better development in the vertical direction. With the distance to the fault decreasing, the overburden breaks, and the collapse angle and the fault inclination are opposite, to facilitate the final establishment of a stable structure. At this time, the effect of the fault on the roof is relatively weak, the fault did not slip significantly and can maintain stability, while the fault near the high-level rock layer deflection to the side of the gob area. When mining to the vicinity of the fault, the fissures in the high-level rock strata are obviously elevated, and eventually, the overburdened rock cannot form an “arch” structure. Eventually, the overburden could not be formed into an “arch” structure. After mining through the fault, initially, due to the fault crushing zone of the absorption, the decompression effect is more significant, so that the working face above the rock layer did not appear a wide range of broken, but with the increase in the distance of the advancement of the direct roof of the layer, the collapsed phenomenon is more obvious, and in the vertical direction continues to develop, the fault on the hanging wall of the overlying rock ultimately formed an “arch” structure, the hanging wall high-level rock layer near the fault plane forms an “inverted triangle” structure and continues to rotate to the gob area^37,38^.

#### Normal stress characteristics of the fault plane

**Figure 8:**
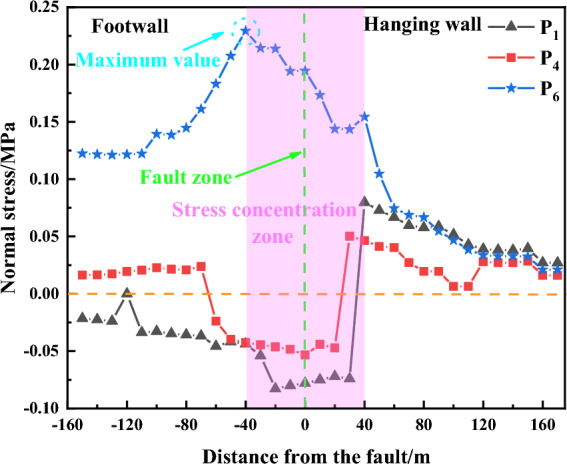
Distribution law of normal stress on the fault plane.

According to the data from the measurement points shown in Fig. 8, P6, P4, and P1 in the monitoring program were analyzed respectively. The strain gauges were first zeroed, with positive sensor readings indicating that they were mainly subjected to pressure, and negative sensor readings indicating that they were mainly subjected to tension.

The distribution of normal stress on the fault plane has obvious spatial characteristics. When far away from the fault, the mining activity of the working face did not play an obvious effect on the stress manifestation of the fault plane, the normal stress can maintain stability, and the normal stress monitored by the three measuring points is approximately equal, but with the decreasing distance from the working face, the P6 stress (the low-level rock layer) continues to grow in the process of subsequent mining, and the change of the low fault zone under the influence of the mining action is relatively significant. In addition, when L = 40–60 m, the growth rate of normal stress is elevated, indicating that the fault plane gradually begins to activate, stress transfer occurs, and in the next mining process normal stress continues to decrease until mining to the fault plane rises again, and ultimately reaches a maximum value of about 0.23 MPa at 40 m through the fault, in the next mining process, the fault at the P6 measurement point is in a fissure. In the following mining process, the fault at measuring point P6 is in the state of fracture closure, and the stress gradually decreases. When L = 40 m, the maximum value of normal stress appears in P6, and in the process of subsequent mining, the normal stress rapidly becomes negative, and the stress value decreases continuously, indicating that the fault at point P6 is in the state of tension at this time, and the unloading of pressure is continuously carried out; Measuring point P4 is advancing in the process of working face advancement, the value of stress is always rising continuously, but the growth rate is lower. When L = 40 to − 40 m, the stress is higher, the recovery to the point 40 m away from the fault, and the stress value is higher. In the range of 40  to − 40 m from the fault, the stress is high, after mining to 40 m from the fault, the fault plane opens up, the normal stress rapidly becomes negative, and in the range of − 40 m from the fault, the fissure closes and turns to positive value; P6 in the high-level rock layer is basically with the trend of P4, but after mining to 40 m from the fault, there is always a fissure in P6, so the normal stress is negative value.

#### Abutment pressure characteristics

**Figure 9:**
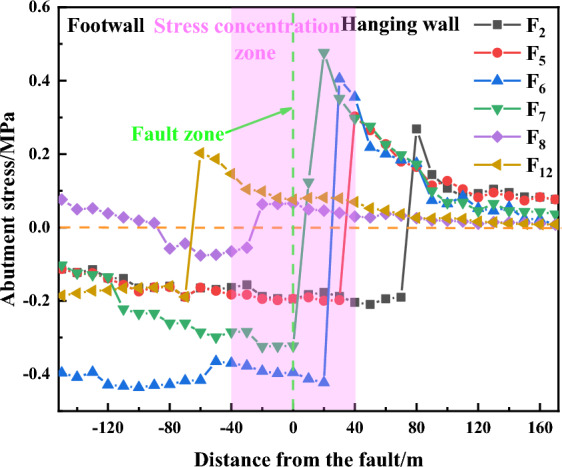
Variation of abutment pressure distribution.

The stress sensor data of F2 (L = 72 m), F5 (L = 30 m), F6 (L = 20 m), F7 (L = 10 m), F8 (L = 10 m) F12 (L = − 58 m) are processed to obtain the study of the distribution law of the abutment pressure as shown in Fig. 9.

In the process of mining, the spatial location relationship between the coal seam and the fault can play an important role in the evolution of the coal body bearing pressure. As the distance between the F2 and F12 measurement points and the fault is relatively far, it makes the abutment pressure distribution by the fault effect is relatively insignificant, after mining through the two measurement points, the two monitoring points’ abutment pressure decreased significantly; as the mining to the fault, the abutment pressure at the F7 monitoring point continues to rise, and at a distance of 20 m from the fault, there is a maximum value of about 0.41 MPa, which is greater than the peak stress value of the other monitoring points, indicating that the working face with a distance of 20 m from the fault is a case of the coal body and fault, the stress peak value is greater than that of the other monitoring points. The maximum value is about 0.41 MPa at 20 m from the fault, which is larger than the stress peak value at any other monitoring point, indicating that in the case that the distance between the working face and the fault is 20 m, the effect of the mining influence between the fault and the working face is more significant, and the abutment pressure is relatively larger. Due to the overlying rock structure damage caused by the mining, the stress field is changed, and the abutment pressure of the working face continues to rise. After the fault plane is opened, the stress cannot be kept continuous, and a high-stress zone appears in the triangular coal pillar area of the fault. After the working face through the fault, the stress at measurement point F8 decreases continuously, the overburdened rock continues to turn back to the gob area, the fissure range of the fault plane expands continuously, and the vertical load on the coal pillar continues to decrease.

#### Characterization of overburden movement

With digital scattering image processing technology, the displacement field of the overburdened rock is studied after the coal seam is extracted, and the displacement field before and after the slip destabilization of the fault is intercepted for comparative analysis.

**Figure 10:**
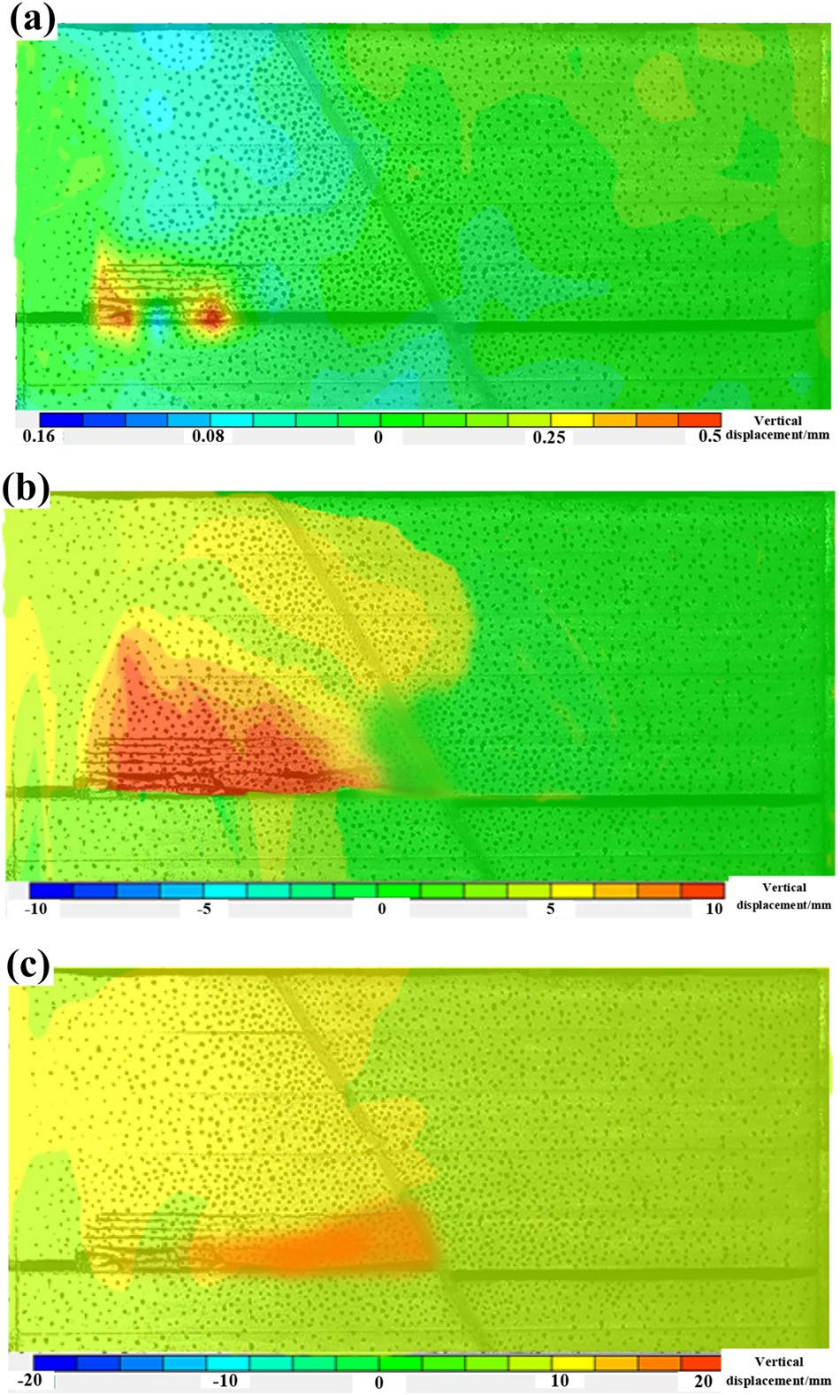
Displacement field variation diagram: (**a**) L = 80 m; (**b**) L = 20 m; (**c**) L = 0 m.

In Fig. 10, as the distance from the fault continues to decrease, the displacement field changes show a certain correspondence with the fault fragmentation zone, and the displacement of the high-level rock layer of the fault is relatively large, which also echoes the similar simulation test in the previous paper, and the activation range of the high-level rock layer continues to develop to the low-level rock layer. After the coal seam is mined from the footwall of the fault, the displacement of the overburden in the vertical direction is relatively large. As the distance between the working face and the fault continues to decrease, the scope of deformation of the surrounding rock in the gob area is significantly increased, and the amount of roof subsidence increases accordingly. Until 20 m away from the fault, the phenomenon of roof fall is more obvious.

When mining to the vicinity of the fault, the extreme value of the rock layer displacement did not change greatly, but the displacement near the fault increased significantly, the displacement of the low-level rock layer increased significantly, and the rock layer movement in the hanging wall of the fault showed irregular changes. After mining through the fault, because the fault has been activated, the rock layer can enter into a stable state again, and the displacement is mainly concentrated in the overburdened rock in the footwall of the fault, indicating that the main roof shows slow subsidence, and the gob area is constantly compacted.

In summary, under the influence of the fault, the continuity of the rock mass has been greatly damaged, and the stress cannot be effectively propagated, showing the characteristic of singularity. When the working face is far away from the fault, the fault does not play a significant role in the distribution of abutment pressure, until L = 30 m, the fault blocking effect is fully shown, the abutment pressure grows rapidly, and the abutment pressure reaches the maximum value when L = 10 m. After the working face mines through the fault, the abutment pressure continues to decrease.

### Results of numerical simulation

#### Failure characteristics analysis of plastic zone in roof

**Figure 11:**
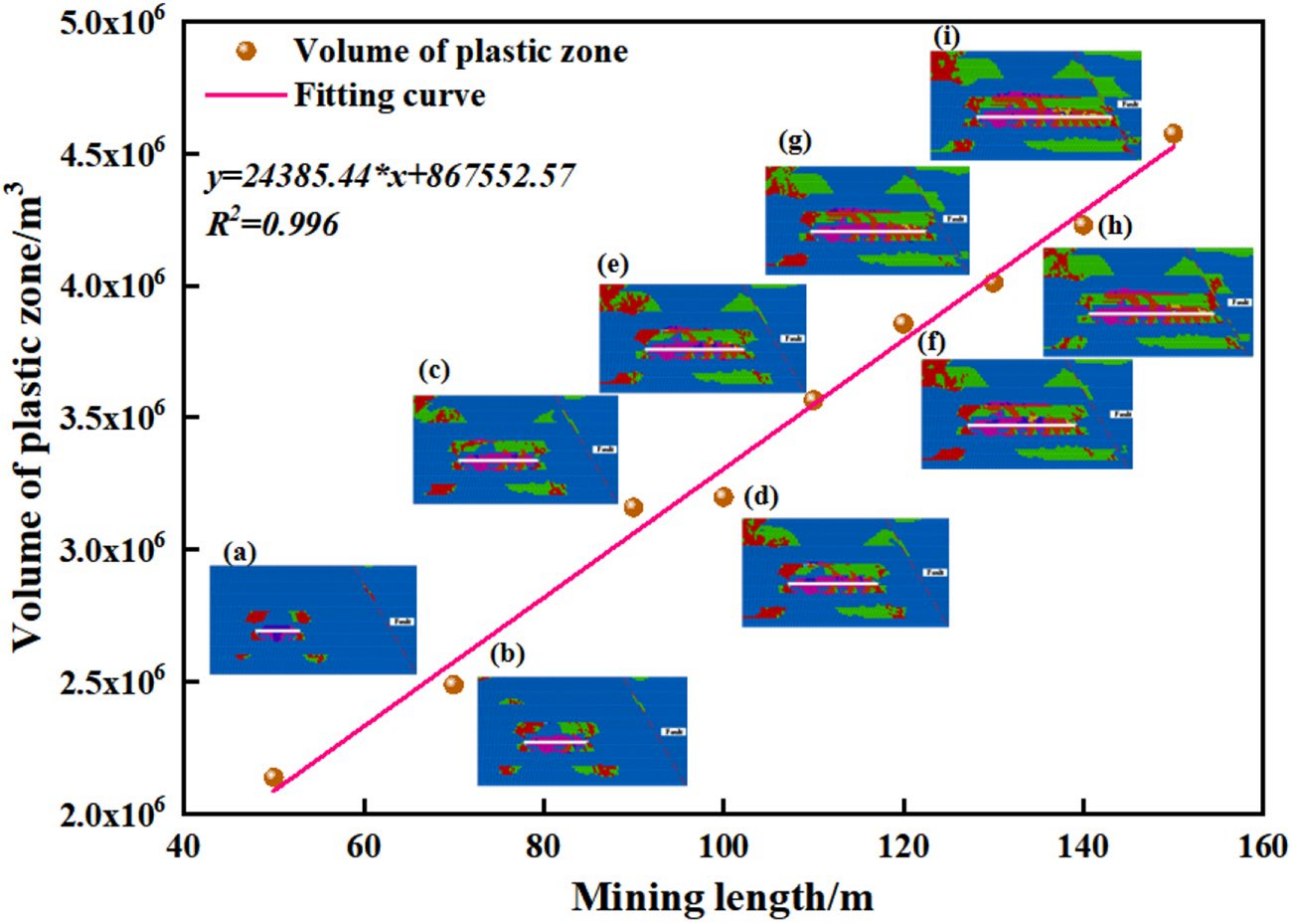
Overall damage volume curves, where (a)–(i) Volume cloud of plastic zone.

As shown in Fig. 11, the plastic zone volume data of 3301 working face at different stages of mining is obtained through fish language, and it can be seen through the fitting curve that there is an obvious positive correlation between the mining length of the working face and the total volume of the plastic zone, and the fitting goodness R^2^ is 0.99, which is excellent, and the corresponding equation of the fitting curve is:

5 $$ {\text{y}} = 867552.57 + 24385.44*x $$

where x is the length of the working face to be mined.

When L ≥ 60 m, the direct roof and direct floor damage is mainly tensile damage, and the main roof and main floor damage is mainly shear damage. The plastic damage of the direct roof and the main roof starts to connect, which indicates that the roof is broken, and the working face has a certain impact danger. In the high-level rock layer around the fault plane, the plastic damage range of the footwall of the fault is relatively large, and the shear damage is dominant, due to the relative independence of the plastic damage of the hanging wall and footwall of the fault, it is considered that the fault is relatively stable at this stage, and it has not entered the activation state.

When 30 ≤ L < 60 m, the plastic damage range of the direct roof and the main roof is connected, and in the high-level rock layer near the fault. The plastic damage range of the footwall is relatively large, and shear damage is dominant. The hanging wall and footwall of the fault are relatively independent of the plastic damage, and shear damage begins to appear in the low-level rock layer, which is considered that the fault gradually begins to activate, and the danger is relatively high.

When L < 30 m, the plastic damage range of the roof continues to increase, and the shear damage range of the hanging wall and the footwall of the fault in the high-level rock layer continues to grow, which mainly occurs in the thicker mudstone; and then the low-level rock layer gradually starts to appear shear damage, and the shear damage range of the direct roof and the direct floor near the fault plane continues to grow. The effect between the fault's footwall and the hanging wall is more obvious, indicating that the fault is fully activated, which seriously threatens the safety production of the working face.

#### Analysis of the stress state of the roof and the results of elastic strain energy

**Figure 12:**
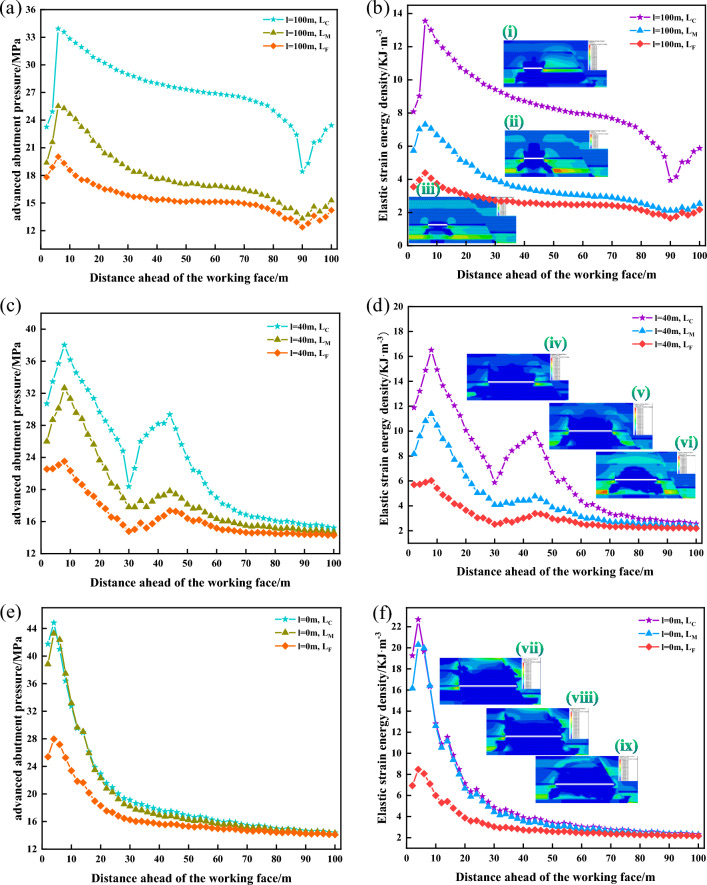
Stress versus energy curves of the roof: (**a**) L = 100 m; (**b**) L = 100 m; (**c**) L = 40 m; (**d**) L = 40 m; (**e**) L = 0 m; (**f**) L = 0 m; where (i)–(ix) is the elastic strain energy density maps.

From Fig. 12, it can be seen that in 3301 working face with different mining lengths, the change rule of roof advance abutment pressure and elastic strain energy density is:

1. The trend of the change of the advanced abutment pressure and elastic strain energy density is consistent, indicating that the advanced abutment pressure plays an important role in the change of elastic strain energy. However, the elastic strain energy accumulated in the hard roof in front of the working face and the peak value of the advance abutment pressure is not strictly coincident, so it can be seen that the elastic strain energy density accumulation is not completely determined by the advance abutment pressure, and the elastic strain energy density accumulation in the direct roof is also affected by the lithological characteristics.
2. At different stages of mining, the advance abutment pressure and elastic strain energy density of the 3301 working face’s roof shows an overall trend of “rapid growth at first, followed by a gradual decrease”, and affected by the tectonic stress and mining stress, the peak occurs near the fault plane, and then continues to decrease.
3. In front of the working face 4–8 m, the advance abutment pressure and elastic strain energy density reached the maximum value, and changed abruptly near the fault, from the initial continuous decrease to the continuous growth, because the fault blocked the propagation of stress, and the superposition of tectonic stress and mining stress resulted in the concentration of stress and energy accumulation on the roof, and eventually the stress and energy distribution on the two sides of the fault were different.
4. The advance abutment pressure of the working face or the elastic strain energy density numerically shows that L_C_ is the highest in the working face. The advance abutment pressure of the working face or the elastic strain energy density numerically shows that L_C_ > L_M_ > L_F_.
5. When the length of the working face is 110 m, the extreme values of the advanced abutment pressure and elastic strain energy density increase rapidly. As the working face is in the “square” range, and the distance between the working face and the fault is 40 m, the mining and tectonic stress is superimposed, putting the roof in a high-stress environment.
6. When the mining length of the working face exceeds 110 m (L < 40 m), the residual bearing stress of the gob area, mining stress, and tectonic stress are superimposed on each other, resulting in the advance abutment pressure and elastic strain energy density of the measuring line L_M_ to rise continuously, and eventually to be consistent with the numerical value of the measuring line L_C_ near the gob area, which indicates that at this stage of the working face, the mining is affected by the impact of the gob area, and the residual bearing pressure in the gob area begins to dominate.
7. The elastic strain energy density cloud diagram of 3301 working face during the mining shows that, with the influence of 3303 gob area, the energy of 3301 working face near the side of the gob area is higher, and there is always a stress concentration area in the floor, and with the decrease of the distance from the fault, the energy accumulation starts to appear in the high-level rock layer near the fault, and the energy decreases rapidly when mining to the fault.

**Figure 13:**
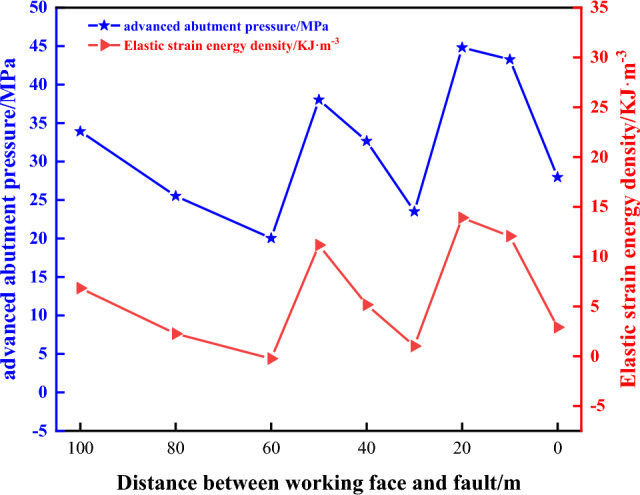
Curve of variation of the extreme value of the elastic strain energy density with the over-head abutment pressure.

Because of the maximum value in Fig. 13, respectively, in the working face mining 100 m and 130 m, at this time, the distance between the fault and the fault was 50 m, and 20 m, respectively, it is considered that the 3301 working face mining to 100 m, it is more significantly affected by the “first square”, and in the subsequent period of the mining process, the pressure strength is relatively weak, and the stress in the roof is transferred to the depth of the rock layer. However, with the decreasing distance from the fault, the stress concentration degree of the working face is higher, and the elastic strain energy density increases. Therefore, it is recommended to strengthen the dynamic monitoring of the mine pressure in these two stages during the mining of 3301 working face and take necessary measures to relieve the pressure if the monitoring is abnormal.

#### Stress evolution and displacement change the law of fault plane

**Figure 14:**
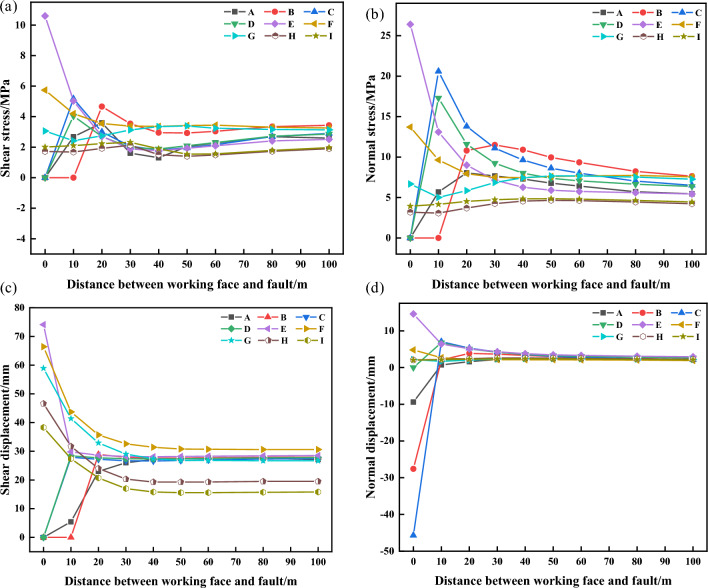
Stress versus displacement curves at fault planes: (**a**) Shear stress; (**b**) normal stress; (**c**) Shear displacement; (**d**) Normal displacement.

From Fig. 14, the stress evolution and displacement change the law of the fault plane is:

1. Each monitoring point on the fault plane has an abrupt change after L ≤ 40 m. This trend is more obvious in the direct roof and direct floor, and in the range of 110–150 m after mining, the shear stress of the monitoring points on the fault plane in the roof decreases to 0, and the monitoring points in the floor all have maximum values. It shows that the activation degree of the fault is relatively high at this time, and when mining to the fault, the shear stress on the fault plane above the coal seam is transferred, and the shear stress below the coal seam is relatively high. Stress relaxation zones appear after the fault plane is damaged, which cannot establish a stable articulation structure, which is not conducive to load transfer, making the coal pillar carry a large number of loads, and the probability of occurrence of dynamic disasters is significantly increased.
2. Whether it is a high-level rock layer or a low-level rock layer, when L ≤ 40 m, the normal stress of the fault plane changes abruptly, and the degree of stress concentration in the high-level rock layer decreases continuously and continues to expand to the low-level rock layer.
3. As the mining of the working face, the fault plane’s equilibrium state is broken, the shear displacement in the overburdened rock layer decreases continuously, and the shear displacement in the underlying rock layer increases continuously.
4. When L > 40 m, the displacement remains unchanged at about 2–3 mm, and when L ≤ 10 m, the normal displacement of the monitoring points arranged on the fault plane near the overburdened rock layer and the coal seam undergoes a sudden change. The direction of normal displacement in the overburden is adjusted, and the normal displacement of the monitoring points near the coal seam is significantly increased.
5. The amount of shear displacement is much larger than the normal displacement, indicating that the activation of the fault plane occurs during the mining, which is mainly affected by the shear stress, and the shear displacement dominates in the fault plane.

#### Basis of fault activation under the influence of mining

Before the mining, the fault is in the stress of primary rock, which can realize the relative stability. The mining to form a series of disturbances gradually acts on the fault, the advanced support pressure changes the initial force equilibrium of the fault plane. When exceeding the fault plane limit equilibrium critical point. Activation instability occurs first in the area of the fault plane with low shear strength, with the mining, the fault plane activation of the “shear” destabilization increased under the common action of the mining stress, residual support pressure of gob area, and the tectonic stresses, and the possibility of the shock induced by the rock burst is significantly increased^7^.

It is found that, for the hanging wall and footwall of the fault, whether it is the high-level rock layer near the fault plane or the low-level rock layer, during the mining of the working face, the shear stress and shear displacement play a dominant role in the activation of the fault.

Therefore, it is considered that the fault starts to enter into the activation state if the plastic damage zone of the high-level and low-level layer of the fault appears to be widely connected and the area formed between the shear displacement curve of the fault plane and the X-axis (the distance between the coal wall of the working face and the fault) is significantly increased compared with the previous stage of mining in the working face.

## Prevention and control of fault rock burst

### Analysis of size retention of fault-protective coal pillar

**Figure 15:**
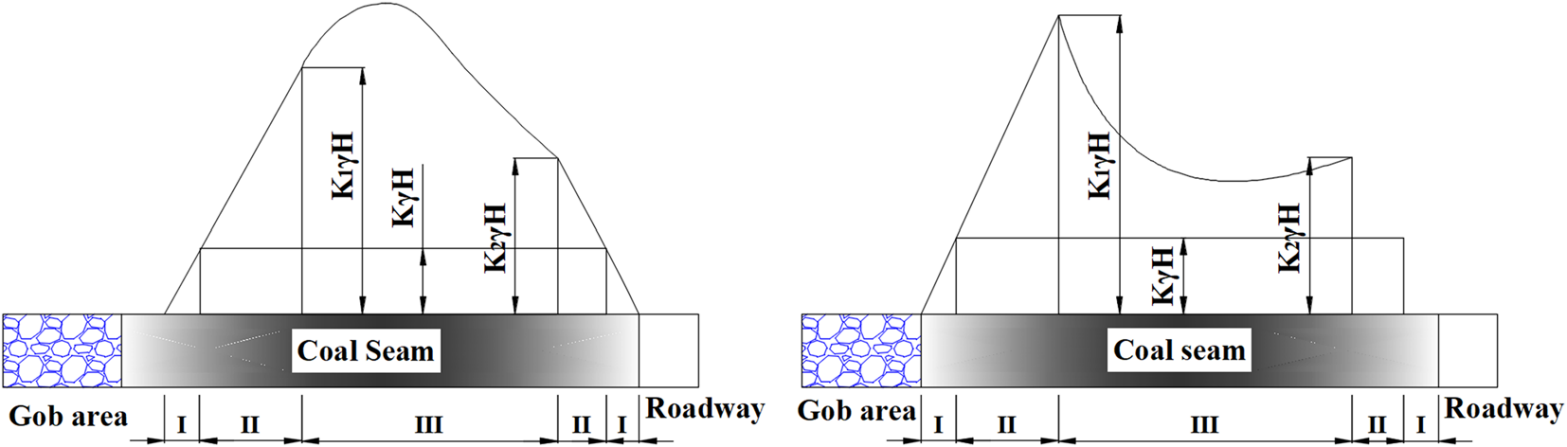
Elasto-plastic zone and stress distribution diagram of one-side mined-out stable coal pillar.

According to the basic geological conditions of the 3301 working face, the coal pillar during the mining is still stable without considering faults: plastic deformation occurs on both sides of the pillar, but its middle position still belongs to the elastic stress zone, the central part of the pillar has a certain range of elastic core^39^, and the width of the pillar can be calculated by the width of the zones, which is shown in Fig. 15.

Among them, *I* is the rupture area, *II* is the plastic area, *III* is the elastic area with elevated stress (elastic core), *L* is the width of the coal pillar, *l*_0_ is the width of the plastic area of the coal pillar on the side of the gob area, *l*_1_ is the width of the plastic area of the coal pillar on the side of the excavated roadway, *γ* is the unit weight rock layer, *H* is the depth of the mining, and *K*_1_, *K*_2_ is the stress concentration factor.

The elastic core range should reach 1–2 times the height of the coal pillar, then the size of one side of the gob area coal pillar can be calculated with the following formula:

6 $$ B = X_{0} + \left( {1\sim 2} \right)M + R $$

where *X*_0_ is the width of the plastic deformation zone on the side of the mining space, m; *M* is the thickness of the coal seam, m; *R* is the width of the plastic deformation zone on the side of the roadway, m.

According to the limiting equilibrium theory of rock mass^40^, the width of the plastic zone of the coal on the side of the mining space of the 3301 working face is *X*_0_:

7 $$ X_{0} = \frac{M}{2\xi f}\ln \frac{K\gamma H + C\cot \varphi }{{\xi \left( {P1 + C\cot \varphi } \right)}} $$

where *K* is the stress augmentation coefficient; *P*_1_ is the resistance of the support to the roadway coal pillar; *M* is the mining thickness of the coal seam; *C* is the cohesion of the coal seam; *φ* is the angle of internal friction of the coal seam; *f* is the friction coefficient of the fault plane between the coal seam and the roof and floor; *ξ* is the triaxial stress coefficient.

Substituting the parameters of Table 4 into Eq. (7), the value of *X*_0_ is calculated to be about 5.08 m.

**Table 4:** Size calculation parameter table of coal body plastic zone at the edge of the mined-out side of 3301 working face.

Symbol	Name	Numerical value	Unit
*K*	Stress augmentation factor	2.50	–
*M*	The thickness of coal seam mined	4.0	m
*P*_1_	Resistance of the support to the coal gang	0.10	MPa
*C*	Cohesion of coal body	2.50	MPa
*f*	Friction coefficient	0.20	–
*φ*	The angle of internal friction of coal body	38	°
*ξ*	Coefficient of triaxial stress	1.18	–
*γ*	Seam bulk weight	25	KN/m^3^
*H*	Mining depth	583.5 (calculated at maximum depth)	m

Similarly, the width *R* of the plastic zone formed near the side of the DF29 fault after the starting cut of the middle roadway of the 3301 working face can be obtained:

8 $$ R = \frac{m\beta }{{2\tan \varphi t}}\ln \frac{{K\gamma H + \frac{C0}{{\tan \varphi a}}}}{{\frac{C0}{{\tan \varphi a}} + \frac{Px}{\beta }}} $$

where *K* is the stress intensification factor, take 1.5; *P*_*x*_ is the resistance of the support to the roadway coal pillar, take 0.1 MPa; m is the height of the roadway, take 3.0 m; *C*_0_ is the cohesion of the coal seam, take 2.5 MPa; *H* is the depth of mining, take 583.5 m; *φ*_*a*_ is the angle of internal friction of the coal seam, take 31.5°; *β* is the lateral pressure coefficient at the interface of the limiting equilibrium area and elastic core, take 0.5, and *γ* is the rock stratum unit weight, take 25 KN/m^3^.

Finally get *R* = 10.5 m, using the formula (6), there is no geological tectonic influence, and the size of the coal pillar B should be more than 23.58 m.

With the mining after moving the working face, the hanging wall of the DF29 fault will also form the gob area, so the coal pillar of the DF29 fault is actually two sides of the gob area, so formula (6) can be changed to:

9 $$ B = 2X_{0} + \left( {1\sim 2} \right)M + R $$

Without considering the DF29 fault, the reasonable width of the coal pillar should be more than 28.66 m. Combined with a similar simulation test and numerical simulation analysis, it is recommended to mine 3301 working face to 20 m away from the fault as a stop-mining line, reserve about 20 m to the protective coal pillar after moving, and finally determine the size of coal pillar of the fault to be 40 m.

### Variable diameter pressure relief drilling technology

**Figure 16:**
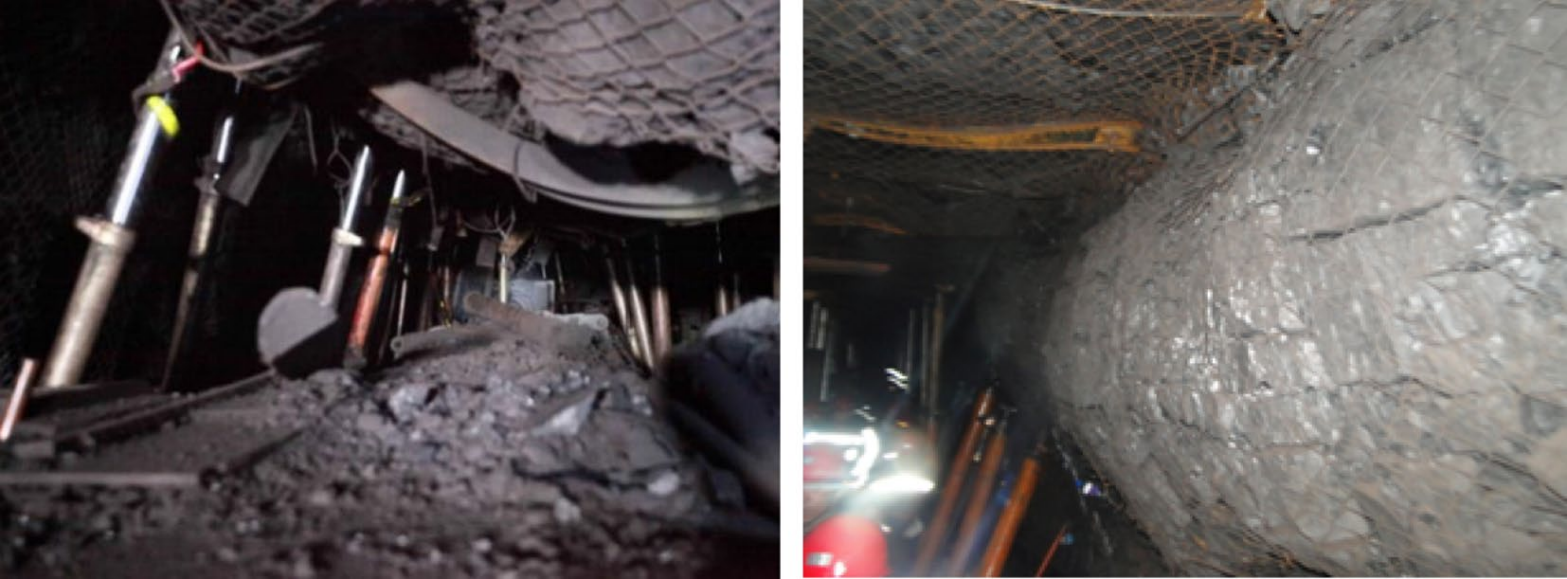
Excessive unloading pressure leads to serious deformation and damage to the roadway site.

On-site construction of large-diameter drilling holes decompression, it is easy to focus on the effect of pressure relief, resulting in the phenomenon of excessive pressure relief and the decompression zone roadway support strength reduction, excessive deformation of the surrounding rock, and roadway section shrinkage is serious (shown in Fig. 16). Aiming at this situation, a variable diameter drilling pressure relief technology is given, to realize the coordinated control of the rock burst and the large deformation of the surrounding rock in the roadway.

**Figure 17:**
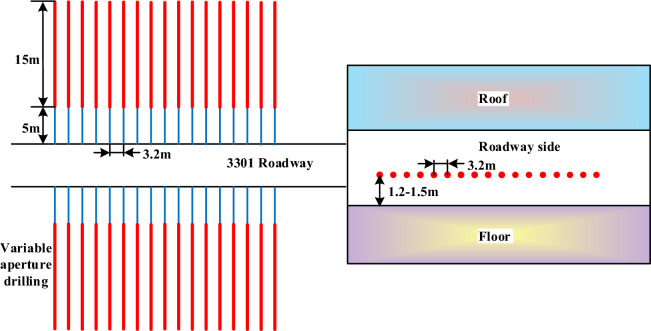
Arrangement of variable diameter unloading borehole.

**Figure 18:**
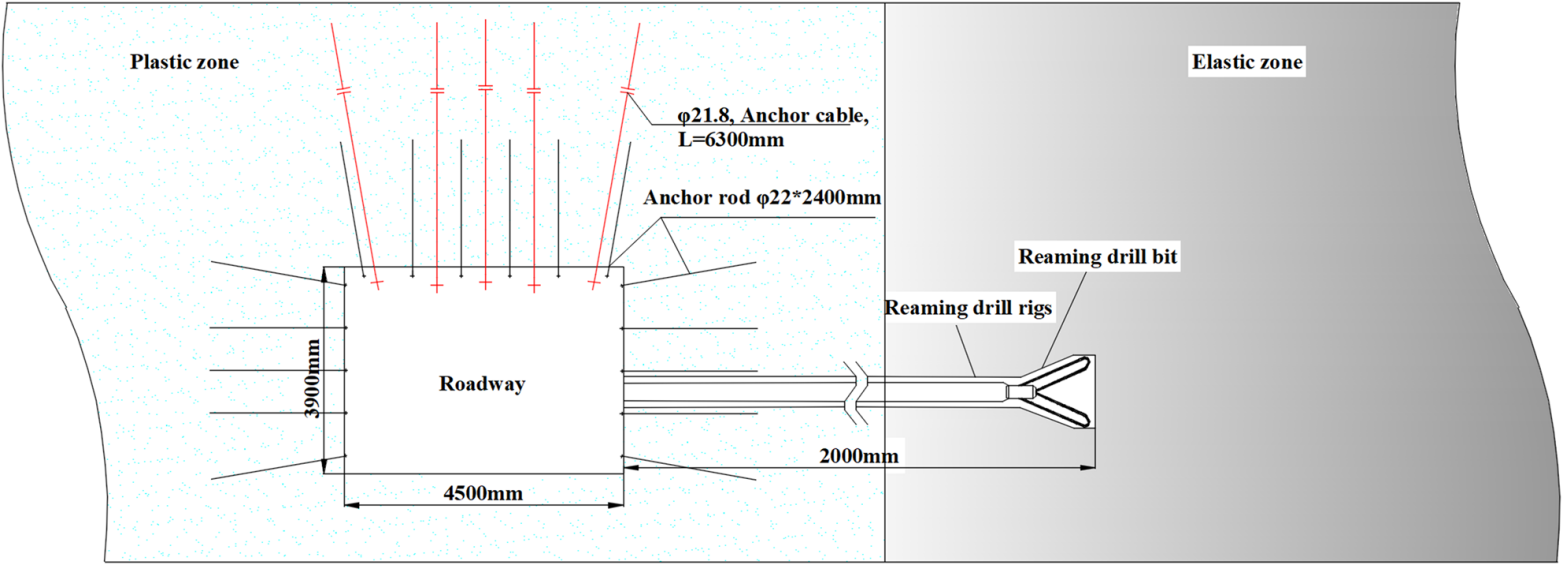
Construction process of variable diameter pressure relief drilling.

1. Drilling rig and drilling tools
  The use of a high-efficiency hollowing drill bit to take measures to reaming and depressurizing in sections, and the drilling equipment for reaming and depressurizing in sections is a CMQS1-450/5.2 S pneumatic drilling truck. The combination of drilling tools is as follows: high-efficiency hole-hollowing drill bit → water-accessible drill pipe → variable diameter joint → drilling truck/drilling rig drive.
2. Drilling arrangement
  According to the previous analysis and research, it is necessary to drill pressure relief holes in the two solid coal roadway sides of the working face, and single-row construction of pressure relief boreholes, with the spacing controlled at 3.2 m, and the distance of the holes from the floor is about 1.2–1.5 m, and the depth of the pressure relief boreholes is 20 m, with the diameter of the outer section of the boreholes (0–5 m) being 90 mm, and the diameter of the section from 5 to 20 m is 240 mm, and the diameter of the outer section of the boreholes (0–5 m) being 90 mm and the diameter of the section from 5 to 20 m is 240 mm. After 5m of drilling, the water is pressurized to open the inner alloy blade of the hollowing bit, and the diameter of the borehole is increased from 90 to 240 mm. The layout plan and section of the variable-diameter unpressurized boreholes are shown in Fig. 17.
3. Construction process
  Determine the reaming position for 5 m, high-efficiency hollowing drill bit drilling to 5m, stop drilling, the nozzles at the blade wings continuously spray high-pressure water., blade continues to drill to the coal seam, and then fully expanded, so that the drill bit can be rotated into the axial pressurized drilling tools to ensure that the cutter wing can be fully stretched, and ultimately realize the rotary reaming. The reaming diameter of 240 mm, and the reaming depth of 15 m in segments, to reach the designated position, will be After reaching the specified position, the drilling tools are orderly withdrawn, and the cutter wings are blocked by the coal seam in the un-reamed position to realize automatic closing, the diameter of the drill bit is restored to 90 mm again, and the drilling tools are withdrawn, so the whole construction process of variable-diameter drilling is completed, as shown in Fig. 18.

### Characteristics of the distribution of microseismic events

**Figure 19:**
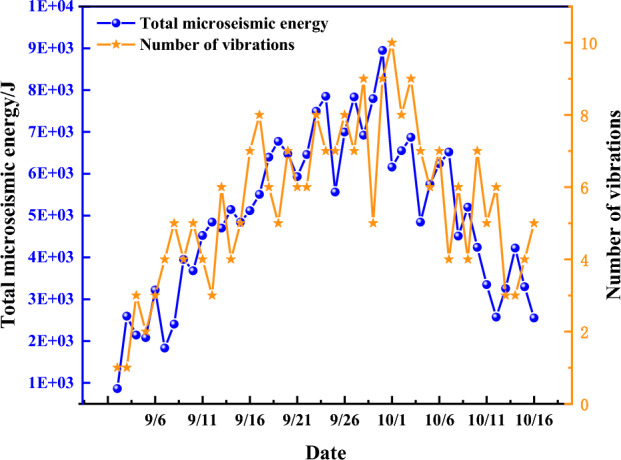
Total energy and frequency of microearthquakes during fault crossing (9.1–10.16).

The evaluation of rock burst has been a hot issue, which can be assessed by evaluating the seismic energy released by the workings mining to the vicinity of the faults^41^. As can be seen from Fig. 19, the impact of mining is relatively small when the working face is kept at a sufficient distance from the fault, and both the number of vibrations and the total energy of microseismicity are significantly elevated as the working face continues to mine forward, and the small-energy events are more concentrated. During the period of crossing the fault, the number of large-energy events increased to a certain extent, and the maximum value of daily microseismic energy was 8947 J, corresponding to a richter scale of 1.65, which further proved the reasonableness of the leaving coal pillar’s size and variable-diameter drilling holes to relief the pressure.

## Discussion

Based on the previous research, this paper makes some innovations on the formation of fault zone^11,16,18^, strictly control the fault dip angle through wooden strips and fine lines, and utilizes digital scattering spots to monitor the displacement of fault overburden rock movement. Through numerical simulation to analyze the elastic strain energy change rule of the roof plate under the influence of the gob area, which is arranged in the working face of the normal fault footwall with different mining distances. The width of the coal pillar protected by the fault and the pressure relief parameters of the variable diameter drill hole are determined in combination with the impact hazard performance during the mining period.

Due to the large differences in the impact hazards of normal faults and reverse faults, even if the burial depth is the same, they still show different characteristics^42^, which leads to the difference in the size of the fault coal pillar and the pressure relief program of the drilling holes^43,44^. This paper mainly focuses on the impact hazard of the 3301 working face under the influence of the mining zone and the lower plate of the normal fault. However, the influence of fault dip angle, fault drop, fault nature, roof strength, and thickness, etc. was not considered. Therefore, more research and practice are needed in the future.

Combined with the fault protection coal pillar formula analysis^39^, it varies according to the buried depth, coal seam mining thickness, and other factors. In addition, the sectional drilling unloading method is mainly applicable to mine production with similar conditions such as large fault drop (more than 20 m) and serious deformation of the traditional large-diameter drilling unloading roadway has a reference role, and it is necessary to appropriately adjust the specific parameters according to the site conditions.

## Conclusion

With the FLAC 3D numerical simulation, studying the changes in the plastic zone and elastic strain energy density of surrounding rock under the influence of 3303 gob area and fault DF29 in the process of 3301 working face mining, and come up with the following main conclusions:

1. With the physical similarity simulation test, found that the footwall working face showed the characteristics of “stable to activation mutation and finally stable” during the mining process towards the fault. The arch structure of the working face was destroyed 20 m away from the fault, and the displacement of the surrounding rock was obviously increased, and the rock around the fault of the high-level rock layer was first activated and the rock around the broken zone was firstly activated. In the case that the working face and the fault are relatively close to each other, the normal stress at the measurement point increases significantly, and the activation range of the high-level rock layer gradually develops to the low-level rock layer, and the activation range of the fault continues to become larger.
2. When mining to 40–60 m from the fault, the growth rate of normal stress increased significantly, indicating that the fault plane gradually began to activate, and in the next mining process, the normal stress continued to decrease until mining to the fault plane rose again, and in the period after crossing the fault, showing a stable growth trend. The predominance of shear displacements on the fault plane and the relatively large displacements in the upper rock strata indicate that there is a relatively high probability of slip in the upper rock strata, which is prone to fault activation.
3. Under the influence of the 3303 gob area, the data of advance abutment pressure and elastic strain energy density near the side of the gob area are relatively high in the process of mining in the 3301 working faces. The maximum value is reached at 4–8 m from the coal wall, and the superposition of tectonic stress and mining stress leads to the concentration of stress and energy accumulation on the roof near the fault.
4. It is considered that the fault starts to enter into the activation state if the plastic damage zone of the high-level and low-level layer of the fault appears to be widely connected and the area formed between the shear displacement curve of the fault plane and the X-axis (the distance between the coal wall of the working face and the fault) is significantly increased compared with the previous stage of mining in the working face.
5. According to the analysis of hazardous area prediction in the process of mining to the working face, it is considered that the impact danger is higher in the two places of 0–30 m and 40–60 m from the fault. The size of the coal pillar of the fault is determined to be 40 m. and combined with variable-diameter pressure relief drilling technology is proposed as a way to achieve coordinated control of impact rock burst and large deformation of the surrounding rock in the roadway.

## Data Availability

All data, models, or codes that support the findings of this study are available from the corresponding author upon reasonable request.
